# The association between healthcare access and shingles vaccination among older adults in Virginia, United States

**DOI:** 10.1371/journal.pone.0316429

**Published:** 2025-04-15

**Authors:** Chidozie Declan Iwu, Pramita Shrestha, Alyson J. Littman, Julia E. Hood

**Affiliations:** 1 Department of Epidemiology, School of Public Health, University of Washington, Seattle, Washington, United States of America; 2 Department of Global Health, School of Public Health, University of Washington, Seattle, Washington, United States of America; 3 Department of Public Health, Kathmandu University School of Medical Sciences, Dhulikhel, Nepal; 4 Department of Veterans Affairs Puget Sound Health Care System, Seattle Epidemiologic Research and Information Center, Seattle, Washington, United States of America; 5 Seattle-Denver Center of Innovation for Veteran-Centered and Value-Driven Care, Health Services, Seattle, Washington, United States of America; 6 Public Health-Seattle & King County, Seattle, Washington, United States of America; Neurocrine Biosciences Inc, UNITED STATES OF AMERICA

## Abstract

**Introduction:**

Shingles is a debilitating vaccine preventable disease that poses a health threat to older adults. However, the uptake of shingles vaccines remains low, and the factors contributing to the low uptake are not clearly understood. This study assessed the association between healthcare access and shingles vaccination among older adults, as well as the impact of COVID-19 pandemic on vaccine uptake.

**Methods:**

This was a cross-sectional study among adults 50 + years in Virginia (n = 16,576) using data from the Behavioral Risk Factor Surveillance System (2018, 2019, and 2021). We calculated the prevalence of shingles vaccination by health insurance and access to primary health care provider (used as proxies for healthcare access) and in relation to the COVID-19 pandemic (pre vs during). Log binomial regression models were used to estimate prevalence ratios (PR), adjusting for confounders.

**Results:**

Shingles vaccination was substantially higher among those with healthcare access compared to those without. Specifically, shingles vaccination was 35% among those with health insurance vs. 10% among those without (adjusted PR (aPR): 2.03, 95% CI 1.44, 2.86), and 36% among those with a primary healthcare provider vs 15% among those without (aPR: 1.99, 95% CI: 1.65-2.41). Finally, shingles vaccination was 41% during the COVID-19 pandemic vs. 30% before (aPR:1.26, 95% CI: 1.20–1.33).

**Conclusion:**

Individuals with health insurance and access to a primary healthcare provider were significantly more likely to receive the shingles vaccine compared to those without such access. Moreover, the prevalence of shingles vaccination during the pandemic period was substantially higher compared with shingles vaccination before the pandemic.

## Introduction

Each year in the United States, about one million individuals are diagnosed with shingles, a painful and debilitating illness caused by the reactivation of the varicella-zoster virus (VZV) [1]. This translates to an incidence rate of 542 to 685 per 100,000 person-years, and approximately 30% lifetime risk of acquiring the disease [2]. The incidence of shingles increases with age, particularly after the age of 50 years. Shingles is typically diagnosed based on clinical symptoms, which include a painful, localized rash that progresses into fluid-filled blisters, often accompanied by burning or tingling sensations. In some cases, individuals may also experience fever, headache, and fatigue [3]. Although shingles typically resolve within a few months, the associated symptoms of painful and itchy blisters can lead to severe long-term complications, such as nerve pain and encephalitis [4]. Management of this condition is costly, with an estimated annual financial burden of $1.3 billion in medical care costs and $1.7 billion in indirect costs in the United States [5].

In the United States, childhood varicella vaccination, introduced in 1995, is now widely administered, with coverage rates exceeding 90% in recent years [6]. This vaccination has significantly reduced the incidence of primary varicella infections, but some studies suggest that reduced exposure to wild-type varicella in adults may lead to decreased natural immunity boosting, potentially increasing the risk of shingles in older adults [7]. Studies have reported an increase in the incidence of shingles over the past two decades in various countries, including the United States, Canada, the United Kingdom, Spain, Japan, Taiwan, and Australia [8–14]. The underlying causes of the observed increase in shingles incidence are still not fully understood, but several hypotheses have been suggested. One possibility is that the availability of antiviral therapy may have increased patients’ willingness to seek care for shingles, therefore increasing diagnosis of shingles. Another possibility is that the widespread use of childhood varicella vaccination may have decreased the natural boosting of immunity from exposure to wild-type VZV. Finally, the increasing use of immunosuppressive therapies for multiple chronic conditions may increase susceptibility to shingles [15].

In response to the growing incidence of shingles, the US Advisory Committee on Immunization Practices (ACIP) recommended the routine use of varicella vaccination for children in 1996 and shingles vaccination for older adults (≥60 years) in 2006 [16]. Two vaccines against shingles, Zostavax (introduced in 2006) and Shingrix (introduced in 2017) have been approved in the United States [17]. The US Centers for Disease Control and Prevention (CDC) recommends that people 50 years and older get two doses of Shingrix separated by 2 to 6 months [18]. These recommendations aim to alleviate the burden of shingles and its associated complications, particularly in high risk populations like the elderly and immunocompromised individuals [19]. Despite these recommendations, the vaccination rate among adults 50 years and older remain low [17]. States in the South Atlantic region including Virginia (34.7%), Florida (28.5%), Georgia (27.2%), North Carolina (32.2%), South Carolina (26.4%), and West Virginia (26.6%) have lower vaccination compared to the US average (35%) [20,21]. Regardless of the state, vaccination rates for shingles is significantly lower compared to the vaccination rates for influenza which was approximately 49% between 2020-2021 according to CDC [22]. In Virginia and other states in the South Atlantic region, the lower uptake of shingles vaccination could be due to socio-economic inequities, highly rural population, and poor healthcare access [17,20,21]. Access to healthcare has a strong and positive correlation with influenza vaccination rates at the state level across diverse population groups [23]. Poor healthcare access constitutes a critical barrier to vaccine awareness, availability, and uptake [24]. Access to healthcare hinges on having health insurance coverage. Individuals without healthcare coverage, including children and non-elderly adults, are less likely to possess a consistent healthcare provider or have had recent medical visits compared to their insured counterparts. In the United States, most individuals under 65 years of age obtain their healthcare coverage from private employer-sponsored group health insurance [25], while nearly all adults aged 65 and above have health insurance through Medicaid or Medicare [26]. Aside from the influenza, pneumococcal polysaccharide, and Hepatitis B vaccines, which are covered by Medicare Part B (included as part of standard medical insurance), all other vaccines, including the shingles vaccine, are covered by Medicare Part D (optional prescription drug coverage that may require an additional premium) for individuals aged 65 years and older [27].

Previous research has shown that healthcare access positively influences vaccination rates for influenza [28] and COVID-19 [29]. However, there remains a gap in understanding how healthcare access affects shingles vaccination among adults. The COVID-19 pandemic introduced unprecedented challenges to healthcare systems, including vaccination efforts [30]. Interestingly, some studies suggest that the pandemic had a positive impact on general vaccination behavior, likely due to heightened awareness of disease risk [31,32]. Yet, there is a scarcity of studies examining the pandemic’s impact on shingles vaccination. To address these gaps, this study aimed to assess the association between healthcare access and shingles vaccination among older adults (≥50 years) in Virginia, and to explore the effects of the COVID-19 pandemic on vaccine coverage. We hypothesize that access to healthcare is a determining factor in Shingles vaccine uptake among older adults in Virginia, United States, and that the COVID-19 pandemic increased the uptake of shingles vaccine. Data from this study will help inform policies and interventions aimed at improving shingles vaccine coverage among older adults.

## Methods

### Study design and data source

We conducted a cross-sectional study utilizing data from the Behavioral Risk Factor Surveillance System (BRFSS) for the years 2018, 2019, and 2021 in Virginia, United States. The BRFSS survey consists of annual telephone interviews that capture health-related risk behaviors, chronic health conditions, and use of preventive services [33]. BRFSS collects data from residents from all 50 states, the District of Columbia, and three US territories, and each state can choose to add additional questions to their surveys. We focused on Virginia because it was the only state that collected data on shingles vaccination across our years of interest. We chose BRFSS because it provides a large population-based, representative sample and allows for state-specific analysis [34].

### Study population

**Fig 1:**
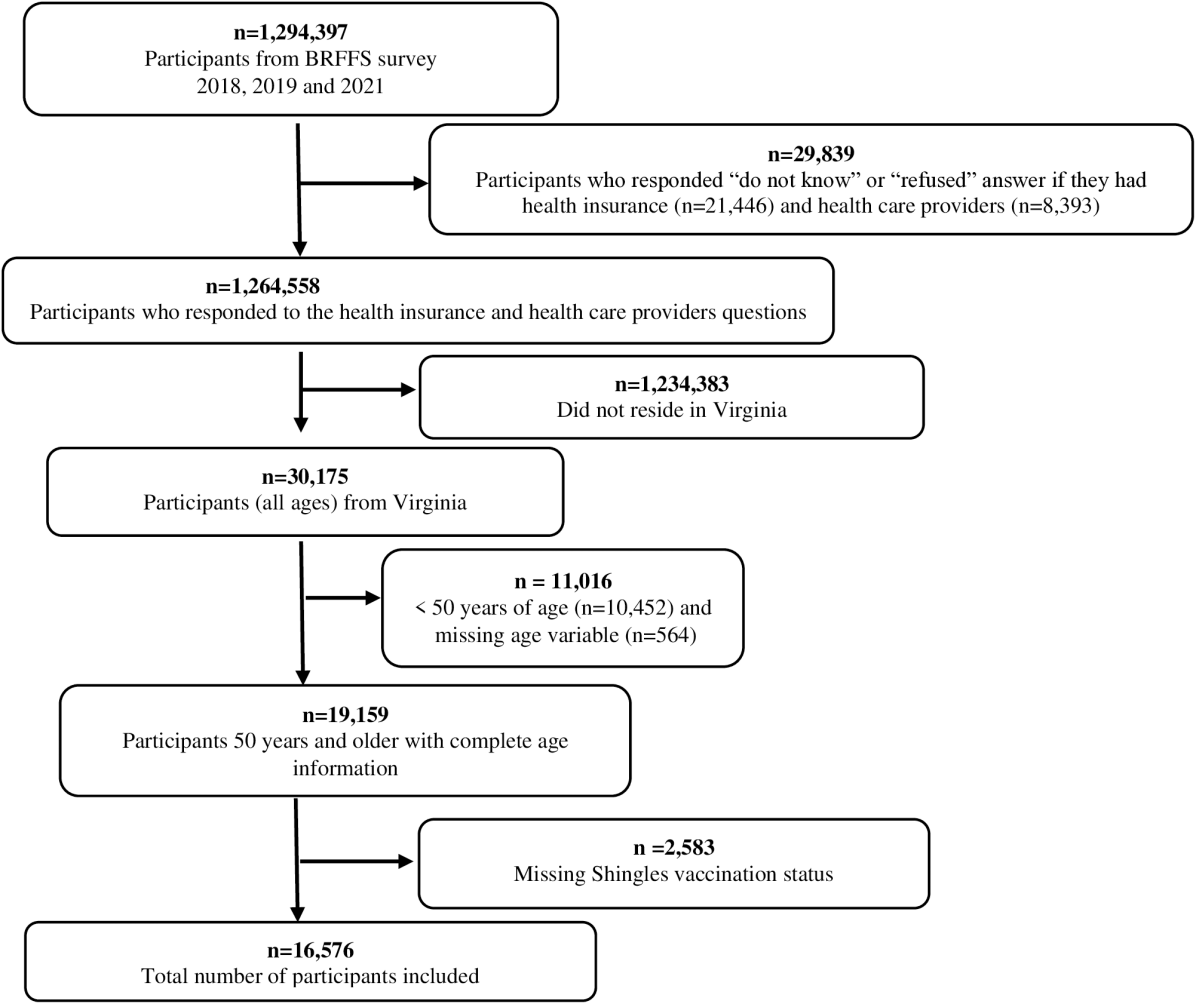
Flowchart of the selection of the study participants.

The study population included adults 50 years and older in Virginia who participated in the shingles module of the BRFSS survey during the years 2018, 2019, and 2021. A total of 16,576 individuals were included in the final analysis (Fig 1).

### Exposures

We used health insurance and access to a primary healthcare provider as primary exposures. These exposures are related to healthcare access but have different constructs. We classified respondents as having health insurance, if they responded “yes” to the question, “*Do you have any kind of healthcare coverage, including health insurance, prepaid plans such as HMOs, or government plans such as Medicare or Indian Health Service?*”. Individuals who responded “do not know” or did not answer the question were excluded from the analysis.

Access to primary healthcare provider was determined based on the question, *“Do you have one person you think of as your personal doctor or healthcare provider?*”. Those who responded “yes, only me” or “more than one” were classified as having access to a primary healthcare provider, while those who responded “no” were classified as not having access to a primary healthcare provider. Those who refused to answer, responded “do not know” or did not answer the question were excluded from the analysis.

The COVID-19 pandemic period was dichotomized as “Pre COVID-19 pandemic” (2018–2019) and “During COVID-19 pandemic” (2021). No information on shingles vaccine was collected in 2020.

### Outcome

Uptake of the shingles vaccination was the outcome of interest. Shingles vaccination was determined based on affirmative responses to, the question, “*Have you ever had the shingles or zoster vaccine*?”.

### Potential confounders

Minimum sets of potential confounders were identified a priori using Directed Acyclic Graphs (DAGs) created by the authors and informed by previous literature [17,35] using DAgitty, version 3.0. This was done to avoid over fitting of the model, ensures a parsimonious model that blocks all non-causal paths, and avoids unnecessary adjustments that could introduce bias or increase variance. For the association between health insurance and shingles vaccination, the minimum set of confounders included age, sex, education, income, rurality and race/ethnicity as shown in S1 Fig in S1 Data. For the association between access to a primary healthcare provider and shingles vaccination, the minimum set of confounders included age, sex, income and race/ethnicity as shown in S2 Fig in S1 Data. For the association between COVID-19 pandemic period and shingles vaccination, the minimum set of confounders included age, sex, race/ethnicity, and rurality as shown in S3 Fig in S1 Data. In this study, we categorized age into two groups as 50–64 years and 65 years and above. Sex was binary variable as male, and female based on the BRFSS question, i.e., “Are you male or female?”. Race/ethnicity was categorized as Black only non-Hispanic, Hispanic, multiracial non-Hispanic, white only non-Hispanic, and another race non-Hispanic. Marital status was grouped as married, unmarried, and never married. Educational status was classified as less than college level, college 1-3 years and college 4 or more years. We grouped income as below $25,000, $25,000–$75,000, and above $75,000. Rurality was dichotomized as urban and rural and employment was classified as employed, retired and unemployed.

### Statistical analysis

Descriptive statistics were summarized to evaluate participant characteristics and to determine the proportion of individuals who reported having access to any health insurance and/or healthcare provider. CDC survey weights were applied to account for BRFSS’s complex survey design and non-response [33]. We evaluated the prevalence of shingles vaccination among the exposure groups. To determine the association between shingles vaccine uptake and access to healthcare, as well as the association between shingles uptake and COVID-19 pandemic periods, log binomial regression models were used to estimate crude and adjusted prevalence ratios and associated 95% confidence intervals. Since approximately 99% of adults aged 65 + years in the United States reported having health insurance [26], a stratified analysis was used to examine if the association between health insurance and shingles vaccination varied between the two age categories (50-64 years and 65 + years). R version 4.2.1 software was used to analyze the data.

### Ethical statement

The University of Washington institutional review board determined that BRFSS analyses do not constitute human subject research. Thus, institutional review board review was not obtained.

## Results

Of the 16,576 individuals who met inclusion criteria, 6,493 individuals indicated having received the shingles vaccine. Table 1 illustrates the characteristics of the BRFSS participants in Virginia stratified by Shingles vaccination status. Most of the study participants (67%) were drawn from the combined 2018 and 2019 BRFSS surveys, compared to 33% from the 2021 survey. Nearly 55% of adults were between the ages of 50-64 years and 45% were 65 years or older. The study had more males (54%) than females (46%). An overwhelming majority reported having health insurance (95%) and access to a primary healthcare provider (91%).

**Table 1:** Characteristics of the BRFSS participants in Virginia stratified by shingles vaccination status and demographics (2018–2019 and 2021).

Characteristics	Vaccinated (n = 6493)		Not vaccinated (n = 10083)		Total (n = 16576)	
	n	Weighted %	n	Weighted %	n	Weighted %
**Years**						
Pre COVID-19 (2018-19)	4015	60	7246	71	11261	67
During COVID-19 (2021)	2478	40	2837	29	5315	33
**Age (years)**						
50-64	1643	34	5670	65	7313	55
65 +	4850	66	4413	35	9263	45
**Sex**						
Female	3786	55	5767	53	9553	54
Male	2707	45	4316	47	7023	46
**Educational status**						
Less than college^a^	1436	27	3502	41	4938	36
College 1-3 years	1571	29	2669	28	4240	29
College 4 or more years	3473	44	3880	31	7353	35
Annual household income status ($)						
Below 25,000	693	11	1993	20	2686	16
25,000–75,000	2102	32	3204	32	5306	32
>75,000	2487	38	3146	31	5633	34
Missing^b^	1211	19	1740	17	2951	18
**Race/ethnicity**						
Black only, non-Hispanic	575	12	1753	21	2328	18
Hispanic	93	3	266	4	359	4
Multiracial, non-Hispanic	78	1	155	1	233	1
White only, non-Hispanic	5538	81	7488	69	13026	73
Another race, non-Hispanic	100	3	218	4	318	4
**Rurality**						
Rural	997	14	1693	14	2690	14
Urban	5496	86	8390	86	13886	86
**Employment**						
Employed	1786	33	4549	51	6338	45
Unemployed^c^	622	11	1703	18	2325	16
Retired	4053	56	3771	31	7824	39
**Health insurance**						
Insured	6367	99	9462	94	15829	95
Uninsured	70	1	522	6	592	5
**Access to primary healthcare provider**						
Yes	6108	96	8703	88	14811	91
No	219	4	1007	12	1226	9

^a^ less than college includes less than a high school graduate, graduated high school or-General Education Development (GED); ^b^missing: only missing values that exceeded 5% were presented; ^c^unemployed: out of work for 1 year or more, out of work for less than a year, self-employed, unable to work, a homemaker, student.

Compared to those who were not vaccinated, a higher proportion of vaccinated respondents were sampled during the COVID-19 pandemic (40% vs. 29%), were 65 years and older (66% vs. 35%), were female (55% vs. 53%), had four years of college or more (44% vs. 31%), were insured (99% vs. 94%), and had access to a primary healthcare provider (96% vs. 88%). Table 2 illustrates the prevalence and prevalence ratios of shingles vaccination by health insurance, access to primary healthcare provider and COVID-19 period. In the adjusted analysis (Table 2), the prevalence of shingles vaccination was about two times higher in those with health insurance versus those without health insurance (adjusted prevalence ratio [PR]: 2.03, 95% CI: 1.44-2.86), and those who had access to a primary healthcare provider versus without access to a primary healthcare provider (adjusted PR: 1.99, 95% CI: 1.65-2.41). The prevalence of shingles vaccination was higher during the COVID-19 pandemic (2021) compared to before the COVID-19 pandemic years (2018-2019) (adjusted PR: 1.26, 95% CI: 1.20-1.33).

**Table 2:** Prevalence ratios of shingles vaccination by health access and COVID-19 pandemic period years among older adults in Virginia, USA (2018–19 and 2021) (n = 16,576).

**Variables**	**Weighted prevalence of shingles vaccination (%)**	**Unadjusted PR**	**95% CI**	**Adjusted PR**	**95% CI**
**Health insurance**					
Uninsured	10	Ref		Ref	
Insured	35	3.52	2.57–4.82	2.03^b^	1.44–2.86
**Access to primary healthcare provider**					
No	15	Ref		Ref	
Yes	36	2.45	2.01–2.99	1.99^c^	1.65–2.41
**Years**					
Pre COVID-19 pandemic (2018–19)	30	Ref		Ref	
During COVID-19 pandemic (2021)	41	1.34	1.27–1.42	1.26^d^	1.20–1.33

PR: prevalence ratio; CI: confidence interval; adjusted PR^b^: adjusted for age, sex, race/ethnicity, income, rurality, and education; adjusted PR^c^: adjusted for age, sex, race/ethnicity, income; adjusted PR^d^: adjusted for age, sex, race/ethnicity, and rurality.

When stratified by age, the prevalence of shingles vaccination was substantially lower among those who were uninsured and 50-64 years (6%) than uninsured and 65 + years (31%). Shingles vaccination rates were higher among those insured, with vaccination rates higher among those 65 + vs. 50-64 (50-64: 22%; 65 + : 50%). When examining the associations between health insurance and shingles vaccination, they were stronger among people 50-64 years old (adjusted PR: 2.70, 95% CI: 1.64-4.46 than among those 65 + years old (adjusted PR: 1.31, 95% CI: 0.85-2.00, Table 3).

**Table 3:** Prevalence ratios of shingles vaccination by health insurance, stratified by two age groups (50–64 and 65 and above) among older adults in Virginia, USA (2018–19 and 2021) (n = 16,576).

Variables	Weighted prevalence of shingles vaccination (%)	Unadjusted PR	95% CI	Adjusted PR^b^	95% CI
**Age group 50–64 years**					
Uninsured	6	Ref		Ref	
Insured	22	3.46	2.47-5.33	2.70	1.64–4.46
**Age group 65 years and above**					
Uninsured	31	Ref		Ref	
Insured	50	1.59	1.05-2.40	1.31	0.85–2.00

PR: prevalence ratio; CI: confidence interval; ^b^adjusted PR: adjusted for race, sex, income, and education.

## Discussion

In this study, we explored the extent to which access to healthcare, including health insurance and access to a primary healthcare provider as well as the COVID-19 pandemic were associated with rates of shingles vaccination among adults aged 50 + years in Virginia. The prevalence of shingles vaccination was twice as high for people with health insurance and access to primary healthcare provider than for those without health insurance or a primary care provider. These findings are consistent with previous findings that have looked at vaccination rates by various factors that comprise the broad definition of “healthcare access” [21], thus shedding light on the disparity of healthcare access. Additionally, the findings contribute to addressing the knowledge gap regarding the impact of healthcare access and the COVID-19 pandemic on shingles vaccination in the older adult population, showing that shingles vaccination was higher during the pandemic compared to in the years prior.

We focused on insurance coverage and primary healthcare providers as proxy measures of healthcare access. These factors have been previously found to be major predictors of healthcare-seeking behaviors [36]. The shingles vaccine can often cost $300 USD or more without insurance, which places a financial barrier that many low-income individuals cannot overcome [37]. Thus, for individuals who may already be struggling economically, the cost of the vaccine makes receiving it unlikely. It is not surprising then, that because shingles vaccination is covered by many health insurance plans [1], vaccination is higher for those who are insured. This emphasizes that individuals without health insurance might face barriers in accessing preventive services like vaccinations, leading to lower vaccination rates. This disparity highlights the importance of making preventive measures more accessible to all individuals regardless of their insurance status, to ensure equitable healthcare outcomes. These findings were consistent across the two age categories of our study participants after stratifying on age and health insurance. However, the association was less pronounced in the 65 + age group, likely because almost everyone in this group possessed some form of health insurance coverage. This underscores the influential role of health insurance in promoting vaccine uptake, highlighting the need to provide preventative care to people younger than 65 years [38]. Similarly, the prevalence of shingles vaccination was higher among those with access to a primary healthcare provider compared to those without. As with insurance, access to a primary healthcare provider has been shown to increase engagement in preventative health services [39]. Primary healthcare providers often act as a main source for addressing health problems and providing encouragement and treatment protocols which a patient can follow. Virginia’s Medicaid expansion in January 2019 likely bolstered healthcare access for those aged 50–64, potentially contributing to the observed increase in vaccination coverage among this demographic. This policy change underlines the critical role of health insurance in fostering equitable access to preventive services, a factor that may have strengthened vaccination rates among Virginia’s lower-income residents [40].

We found that the prevalence of shingles vaccination was higher during the COVID-19 pandemic than a few years prior. This corroborates a previous study [32] suggesting that the heightened awareness of disease risk and the increased focus on vaccinations during the pandemic may have positively influenced the uptake of other recommended vaccines, such as the shingles vaccine, in older adults. Although we cannot infer causality due to the cross-sectional nature of this study, several factors including public awareness campaigns, healthcare provider engagement and integration with COVID-19 vaccination effort may have driven the results we found. Those disproportionately affected by COVID-19, the elderly and immunocompromised, are also the same demographic which shingles tend to affect the most. Thus, because COVID-19 hospitalization and mortality were particularly high among elderly individuals, the perceived susceptibility to vaccine preventable illness may have increased among older adults, leading to higher vaccination rates among that population. These insights underscore the interconnectedness of health crises and the potential for public health responses to have far-reaching positive impacts. One study observed an increased risk of shingles following COVID-19 vaccination, which provided some evidence of the complex interplay between pandemic, vaccination for COVID, and vaccination for other illnesses. [41].

## Limitations and strength

This study has several limitations. Firstly, our definition of healthcare access did not consider other types of healthcare access, providing a limited view of the complexities of healthcare. Nonetheless, these factors have been found to be important indicators of healthcare access. Secondly, the relatively small number of people without health insurance and access to primary healthcare makes this study only generalizable to older people. Further, the cross-sectional nature of our study limited causal inference; temporality cannot be determined and claims about causality cannot be established. Key exposures and outcomes were based on self-reported data, which may introduce biases such as social desirability, recall bias, and nonresponse. An important strength of our study was the large sample size, which allowed us to have enough statistical power to infer the associations of interest. This study used BRFSS data, a well-established and widely used survey that captures health-related risk behaviors and preventive service utilization. This ensures that the findings are representative of the population in Virginia. To the best of our knowledge, this is a first study that assessed the impact of the COVID-19 on shingles vaccination among older adults. This study also provides insights into how health disparity impacts preventive measures such as vaccinations.

## Conclusion and recommendations

The prevalence of shingles vaccination was higher among individuals with better healthcare access and during (vs. before) the COVID-19 pandemic. The association between health insurance and shingles vaccination was stronger among those 50–64 years old compared to 65 + years old. Understanding the link between healthcare access and vaccination rates can guide targeted efforts to increase vaccination uptake among vulnerable populations. It might prompt initiatives to improve vaccine equity among older adults. The findings of this study could also guide the development of policies and interventions aimed at enhancing vaccination efforts regardless of the presence of public health emergencies. We recommend that future studies incorporate further healthcare access indicators for comprehensive understanding and conduct a longitudinal study to understand the long-term impact of public health emergencies on other health conditions.

## Supporting information

S1 Data The Directed Acyclic Graph for the association between health insurance and shingles vaccination (minimum set of confounders: age, income, education, location/rurality, and race/ethnicity). S2. The Directed Acyclic Graph for the association between health care provider and Shingles vaccination (minimum set of confounders: age, race/ethnicity and income). S3. The Directed Acyclic Graph for the association between COVID period and Shingles vaccination (minimum set of confounders: age, race/ethnicity, rurality, and sex).
(DOCX)
