# Supplementary material for: The association between healthcare access and shingles vaccination among older adults in Virginia, United States
Source: PLoS One. 2025 Apr 15;20(4):e0316429. doi: 10.1371/journal.pone.0316429 (PMC11999105; doi:10.1371/journal.pone.0316429)
Supplement: S1 Data — S2. The Directed Acyclic Graph for the association between health care provider and Shingles vaccination (minimum set of confounders: age, race/ethnicity and income). S3. The Directed Acyclic Graph for the association between COVID period and Shingles vaccination (minimum set of confounders: age, race/ethnicity, rurality, and sex). (DOCX) [file pone.0316429.s001.docx]

**SUPPLEMENTARY FIGURES**


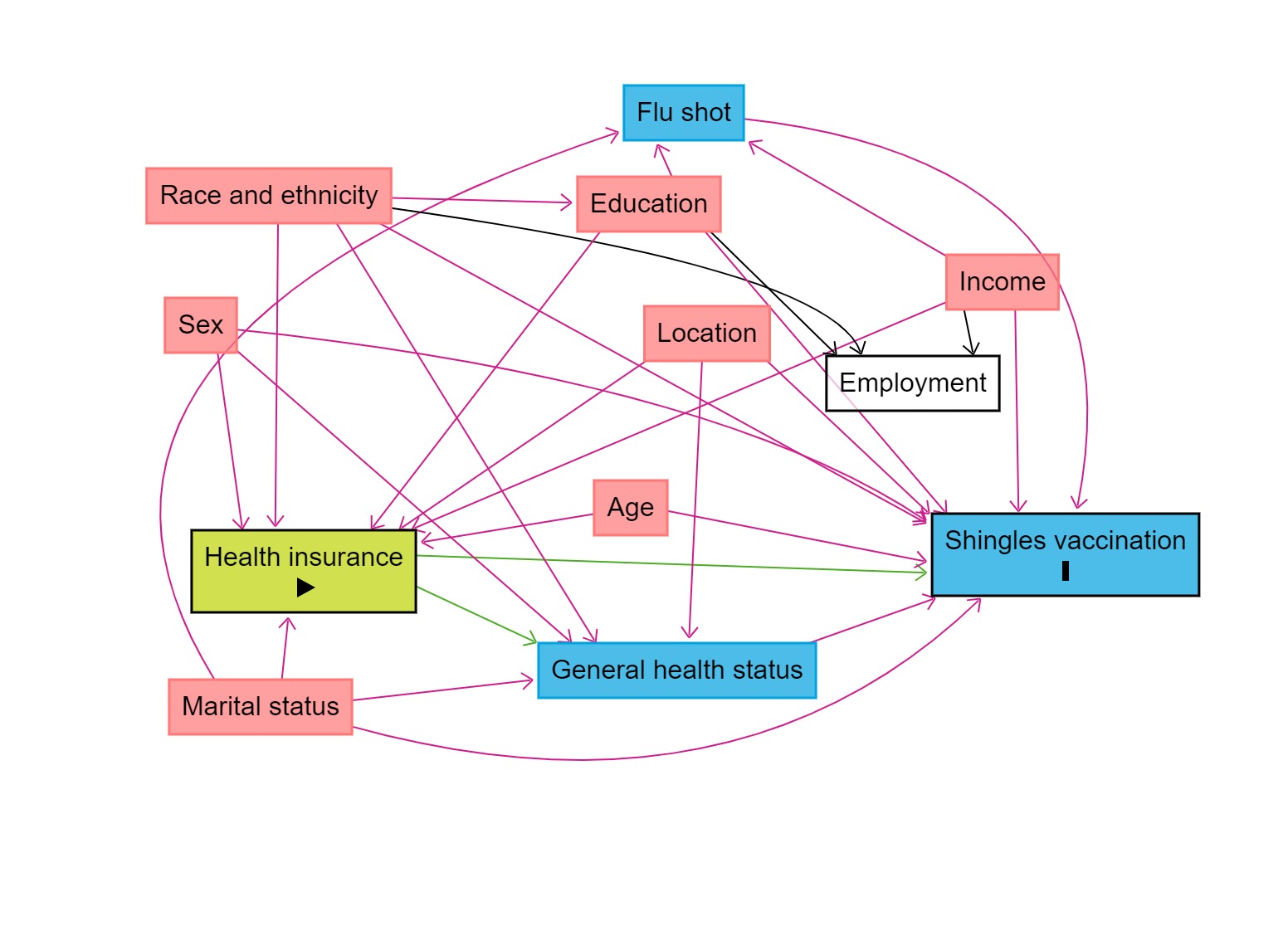

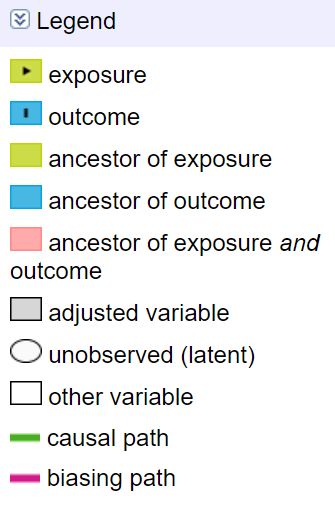


**Supplementary Figure 1:** The Directed Acyclic Graph for the association between health insurance and Shingles vaccination (minimum set of confounders: age, income, education, location/rurality), and race/ethnicity)


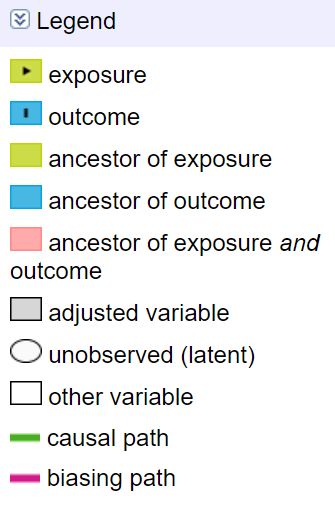

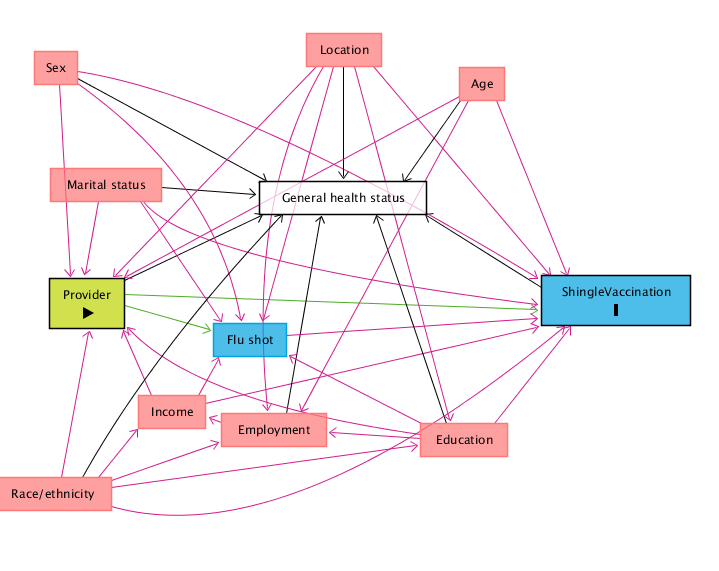


**Supplementary Figure 2:** The Directed Acyclic Graph for the association between health care provider and Shingles vaccination (minimum set of confounders: age, race/ethnicity and income).


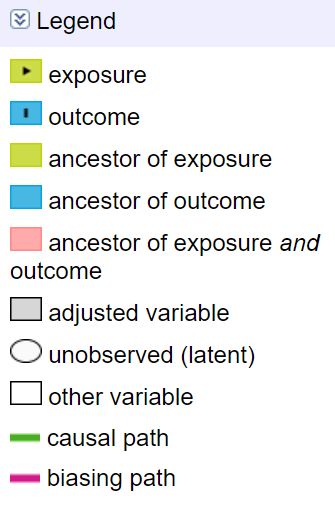


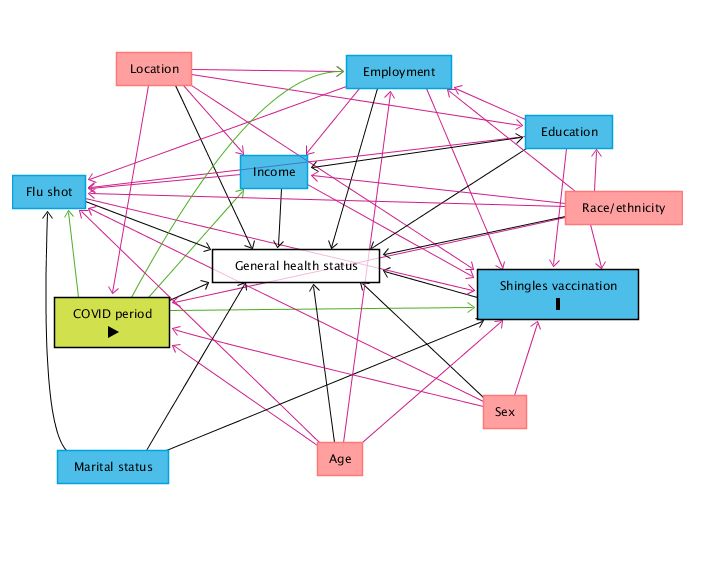


**Supplementary Figure 3:** The Directed Acyclic Graph for the association between COVID period and Shingles vaccination (minimum set of confounders: age, race/ethnicity, rurality, and sex).
